# A Century Searching for the Neurons Necessary for Wakefulness

**DOI:** 10.3389/fnins.2022.930514

**Published:** 2022-07-19

**Authors:** Fillan S. Grady, Aaron D. Boes, Joel C. Geerling

**Affiliations:** ^1^Geerling Laboratory, Department of Neurology, Iowa Neuroscience Institute, The University of Iowa, Iowa City, IA, United States; ^2^Boes Laboratory, Departments of Pediatrics, Neurology, and Psychiatry, The University of Iowa, Iowa City, IA, United States

**Keywords:** brainstem, wakefulness, arousal, coma, ascending reticular activating system

## Abstract

Wakefulness is necessary for consciousness, and impaired wakefulness is a symptom of many diseases. The neural circuits that maintain wakefulness remain incompletely understood, as do the mechanisms of impaired consciousness in many patients. In contrast to the influential concept of a diffuse “reticular activating system,” the past century of neuroscience research has identified a focal region of the upper brainstem that, when damaged, causes coma. This region contains diverse neuronal populations with different axonal projections, neurotransmitters, and genetic identities. Activating some of these populations promotes wakefulness, but it remains unclear which specific neurons are necessary for sustaining consciousness. In parallel, pharmacological evidence has indicated a role for special neurotransmitters, including hypocretin/orexin, histamine, norepinephrine, serotonin, dopamine, adenosine and acetylcholine. However, genetically targeted experiments have indicated that none of these neurotransmitters or the neurons producing them are individually necessary for maintaining wakefulness. In this review, we emphasize the need to determine the specific subset of brainstem neurons necessary for maintaining arousal. Accomplishing this will enable more precise mapping of wakefulness circuitry, which will be useful in developing therapies for patients with coma and other disorders of arousal.

## Introduction

Consciousness is central to the human experience, and wakefulness is a necessary prerequisite for consciousness. Lack of wakefulness manifests as coma, which is distinguished from sleep by a lack of arousability. Many intermediate states exist on a spectrum from severe impairments like coma and stupor to alert wakefulness. There are no efficacious treatments for severe impairments in consciousness, and supportive care with treatment of underlying medical conditions remains the standard of care (Posner et al., 2007).

Our understanding of the brain circuits that sustain wakefulness remains incomplete, which impedes rational therapeutic development. Therefore, understanding the specific neural circuits that produce and maintain arousal is crucial for advancing the neuroscience of consciousness and identifying new therapeutic opportunities.

Over the last century, parallel lines of evidence have identified a brainstem region that is necessary for wakefulness. Lesion studies in experimental animals and in human patients have converged on a region of the upper brainstem tegmentum where damage causes coma. This region is the only location in the nervous system where a single, focal lesion can cause coma. It contains genetically diverse neurons with widely varying patterns of connectivity, but it remains unclear which are necessary for wakefulness.

In this article, we will consider whether neurons of interest satisfy two criteria. First, are they necessary for arousal, such that silencing or ablating the neurons eliminates or greatly diminishes wakefulness? Second, does stimulating the neurons produce (or prolong) wakefulness?

Lesion experiments, which test necessity, have focused attention on a region of the pons and midbrain tegmentum including the locus coeruleus, parabrachial nucleus, and many other populations of nearby neurons (Bremer, 1935; Lindsley et al., 1949; Rossi and Zirondoli, 1955; Batini et al., 1958; Jones et al., 1977; Kerkhofs and Lavie, 2002; Fuller et al., 2011). Stimulating neurons in this region causes experimental animals to awaken from sleep, to remain awake longer than usual, or to awaken more rapidly from anesthesia (Moruzzi and Magoun, 1949; French and Magoun, 1952; Carter et al., 2010; Muindi et al., 2016; Qiu et al., 2016; Kroeger et al., 2017; Wang et al., 2019). However, sufficiency for promoting arousal is not evidence that a particular set of neurons is necessary for sustaining arousal. As a counterexample, activating a variety of brain regions and even peripheral nerves has a similarly arousing effect (Bonvallet et al., 1954; Rothballer, 1956; Candeias da Silva et al., 2022). Thus, in this review, we focus more on necessity as the standard of evidence in considering which regions and which specific neurons sustain arousal. In doing so, we argue that an unidentified population of neurons in the upper brainstem tegmentum is necessary for sustaining arousal. We will also discuss downstream targets in the forebrain but will focus primarily on the brainstem.

This review will also discuss pharmacological manipulations, such as infusing an exogenous neurotransmitter, receptor agonist, or receptor antagonist into the body. These drugs are sometimes microinjected into specific regions of the brain but are more frequently injected into the cerebrospinal fluid or even into the circulatory system, which compromises anatomical specificity.

Before reviewing key studies leading to our current knowledge in this area, it is important to clarify how investigators measure wakefulness. Readily observable behavioral correlates of wakefulness include spontaneous eye opening and meaningful, non-reflexive engagement with the environment. However, lesions that disrupt wakefulness also have the potential to disrupt these behavioral correlates and confound the assessment of wakefulness. Work in this area of neuroscience accelerated after the availability of electroencephalography (EEG), which measures electrical activity in the cerebral cortex as a surrogate measure of arousal. While cortical EEG signals are an imperfect correlate of clinical arousal (Westmoreland et al., 1975; Halder et al., 2021; Baron and Devor, 2022), the amplitude and frequency of cortical EEG signals can be combined with video recordings and electromyography (EMG) as a marker for states of wakefulness, slow wave sleep, and rapid-eye-movement (REM) sleep.

## Animal Experiments

**FIGURE 1:**
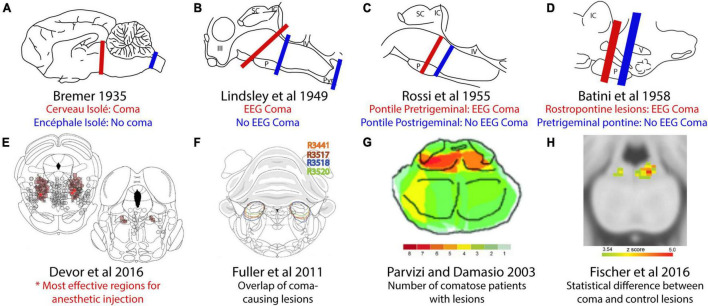
Comparison of coma-causing and non-coma-causing lesions in the brainstem. **(A)** Bremer’s *cerveau isolé* midbrain transections caused coma but *encéphale isolé* transections between the brainstem and spinal cord did not. **(B)** Later, rostral pontine transections caused coma, but more caudal transections did not. **(C)** An iterative search for the coma-causing region identified a small region of the rostral pons. **(D)** Transections at a different angle identified the importance of the pontine tegmentum. **(E)** Micro-injection of the anesthetic pentobarbital produced anesthesia at the lowest doses in the pons-midbrain tegmentum. **(F)** Orexin-saporin lesions of the parabrachial region caused coma. **(G)** Overlap analysis of patients with coma-causing lesions identified a similar area of the human brainstem. **(H)** Subsequent comparison of coma-causing versus control lesions identified a more focal region of the human pontine tegmentum. This figure adopted from Devor and Zalkind (2001).

The rigorous experimental study of arousal began with the work of Bremer (1935). He combined axial transections in the midbrain, which he termed the “cerveau isolé” preparation, with EEG recordings in cats. Bremer’s transections produced dramatic results, causing the EEG to develop highly synchronous waves, similar to the EEG appearance during sleep. Later scientists would observe that some animals with midbrain transections gradually regained cortical desynchronization and circadian rhythms over the next month (Lindsley et al., 1950; Villablanca, 1962, 1966; Slosarska and Zernicki, 1973), mirroring the clinical course of human patients who survive coma-causing strokes in the pons-midbrain tegmentum (Posner et al., 2007). In contrast, Bremer’s “encéphale isolé” transections (Figure 1A) between the brain and spinal cord left intact EEG rhythms reflecting normal sleep-wake cycles. He hypothesized that the brain required ascending sensory input to remain awake, and that the caudal “encéphale isolé” lesions left enough cranial nerve afferents intact to maintain wakefulness (Bremer, 1935; Kerkhofs and Lavie, 2002).

Lindsley et al. (1949) continued Bremer’s work by making brainstem transections and electrolytic lesions in cats. These lesions interrupted ascending fibers of passage at three different rostrocaudal levels, and in agreement with Bremer’s work, cats with transections in the upper cervical spinal cord or caudal pons had EEG rhythms reflecting a normal sleep-wake cycle. In contrast, lesions at or rostral to the pons-midbrain junction produced low-amplitude, synchronized rhythms, indicative of coma (Figure 1B). They followed this up with electrolytic lesions, which produced EEG rhythms reflecting coma after destruction of the midbrain tegmentum, but not in cases with destruction of the lateral and dorsal parts of the midbrain.

In a complementary approach, Moruzzi and Magoun (1949) identified a zone stretching from the medulla to the thalamus where electrical stimulation produced EEG signs of arousal in cats. Electrolytic lesions in the midbrain tegmentum abolished this effect, whereas lesions in the lateral and ventral midbrain did not. From this work, they concluded that they were not stimulating the lemniscal sensory pathway, but instead a parallel sensory pathway to the thalamus, which they termed the “ascending reticular activating system” (Starzl et al., 1951; Magoun, 1952). Despite their historical significance, these experiments relied on crude stimulation of large parts of the brainstem. These stimulation experiments used high voltage (3V, not constant current) at frequencies of 50–300 Hz for several seconds, far faster than the firing rate of most brainstem neurons (Saito et al., 1977; Sieck and Harper, 1980; Gilbert and Lydic, 1990; Steriade et al., 1990; Sakai, 2017).

A Japanese group identified a similar region that, when damaged, caused coma. They focused on the brainstem after a human patient became comatose following an “exploratory puncture.” They attempted to verify this finding by experimentally puncturing the brainstem in awake cats and monkeys with glass rods. When these rods unilaterally damaged the pontine tegmentum medial to locus coeruleus, the animals quickly became comatose. These findings have not received much attention but also point to an area in the upper brainstem tegmentum as necessary for wakefulness (Tsubokawa and Katayama, 1985).

Subsequent investigators again used transections to narrow the search for the brainstem level necessary for wakefulness. Systematically varying the rostrocaudal level revealed that transections immediately rostral to the trigeminal nerve roots caused coma, whereas transections immediately caudal to them did not (Figure 1C; Rossi and Zirondoli, 1955) and they hypothesized that trigeminal input is necessary for wakefulness. Batini et al. (1958, 1959) made electrolytic transections at a slightly different angle, separating the trigeminal nerve roots from the brain while retaining more of the pons-midbrain tegmentum dorsally. These did not cause coma, but placing a second lesion rostrally caused coma, as predicted (Figure 1D). The authors concluded that the “integrity of a small amount of nervous tissue lying in the rostral part of the pons,” rather than trigeminal input, maintains wakefulness.

Other investigators focused on the ascending pathways that carried the output of these arousal-producing nuclei to the forebrain. Building on the stimulation results of Magoun and colleagues, Jones et al. (1973) placed large electrolytic lesions in the ventral tegmentum and in the “nebula complex,” a region that includes the A8 catecholamine neurons and central tegmental tract, which carries the axonal projections of the locus coeruleus (Jones and Moore, 1977a) and parabrachial (Saper and Loewy, 1980) neurons to the cerebral cortex. Ventral tegmental lesions caused akinesia, while “nebula complex” lesions in cats decreased wakefulness over the following 2 weeks, with some animals spending as little as 8% of their time awake. The authors hypothesized that ventral tegmental dopaminergic neurons controlled movement, and ascending noradrenergic fibers were responsible for cortical arousal.

Lidbrink (1974) attempted to more selectively destroy catecholaminergic axons in the central tegmental tract by using a toxin, 6-hydroxydopamine (6-OHDA). He injected this toxin into the central tegmental tract (which contains the dorsal noradrenergic bundle), at two different rostrocaudal levels (Lidbrink, 1974). These lesions, regardless of rostrocaudal level in the central tegmental tract, caused a loss of cortical norepinephrine with a temporary decrease in active wakefulness and a corresponding increase in quiet wakefulness for the following week. The amount of active wakefulness correlated with cortical norepinephrine, with some rats only spending 7% of their time in active wakefulness. However, these rats were arousable and grossly normal when stimulated by the experimenter.

Together, these experiments raised the idea that noradrenergic axons carried by the central tegmental tract from the locus coeruleus to the cortex are necessary for normal levels of arousal (Jones and Moore, 1977a). Importantly, and in contrast to larger brainstem lesions, neither approach resulted in coma; animals remained grossly normal and arousable by the experimenters.

Later experimenters again used electrolytic lesions to narrow down the region of the brainstem necessary for arousal. Jones et al. (1977) placed stereotaxic, electrolytic lesions in the pons tegmentum. Electrolytic lesions in the medial pontine and midbrain tegmentum decreased time spent awake. Conversely, lesions in the dorsolateral pontine tegmentum produced a transient coma, lasting 12–48 h. The cats were unresponsive to stimuli and their respiratory rates initially declined to 2–3 breaths/minute. Their apneustic breathing raises the possibility that these lesions damaged the “pontine pneumotaxic center” (Lumsden, 1923; Chamberlin and Saper, 1994) causing a respiratory encephalopathy that may have secondarily influenced arousal.

Later, Devor and Zalkind (2001) microinjected the GABA-A receptor agonist pentobarbital into the pons-midbrain tegmentum, causing reversible coma in rats. They found that a region in the caudal midbrain tegmentum required the lowest dose (Figure 1E; Devor and Zalkind, 2001). It seems likely that anesthesia transiently silenced a group of neurons necessary for wakefulness, and that these are the same neurons that, when silenced, produce the behavioral effects described by Bremer and others. Interestingly, however, while other GABAergic agents produce loss of arousal, neither silencing this region with lidocaine or tetrodotoxin (Devor et al., 2016; Minert et al., 2017; Avigdor et al., 2021), nor ablating it caused loss of arousal (Minert and Devor, 2016; Lanir-Azaria et al., 2018). No specific cell populations have been identified as mediating the effect of pentobarbital in this region (Minert et al., 2017).

Fuller et al. (2011) proposed that the neurons necessary for wakefulness are located caudal to this area, in the parabrachial nucleus (PB). Their injections of saporin (a ribosomal toxin) conjugated to the neuropeptide orexin disrupted much of the PB region and caused coma in rats (Figure 1F; Fuller et al., 2011). Conversely, disruption of surrounding brainstem structures, such as the cholinergic laterodorsal tegmental nucleus, locus coeruleus, and the dorsal raphe did not reduce wakefulness (Lu et al., 2006b; Fuller et al., 2011). Although the intended purpose of orexin-saporin was to selectively ablate neurons that express hypocretin/orexin receptor, this toxin non-specifically destroyed neurons in the region (Fuller et al., 2011). Therefore, the precise neuronal populations and boundaries of the zone necessary for wakefulness remain unclear.

Other pieces of evidence point to a role for the PB in arousal. On the sufficiency side, chemogenetic activation of neurons in this region caused several continuous hours of wakefulness (Qiu et al., 2016), and chemogenetic activation of the *Calca*-expressing subset of PB neurons modestly increased wakefulness (Kaur et al., 2017). Also, stimulation of the parabrachial nucleus with electrodes (Muindi et al., 2016), and with chemogenetic or optogenetic tools (Luo et al., 2018; Wang et al., 2019) increased the speed with which mice awaken from general anesthesia.

The necessity of the PB for maintaining wakefulness is less clear. To prevent the release of glutamate, which is the fast excitatory neurotransmitter of PB neurons, Kaur et al. (2013) deleted *Slc17a6* (Vglut2) from this region. While this manipulation reduced hypercapnic arousal, baseline wakefulness was only mildly impacted (Kaur et al., 2013). Further, the PB has been lesioned bilaterally in many experiments involving gustatory discrimination, conditioned taste aversions, and breathing, with no reports of coma (Reilly et al., 1993; Spector et al., 1993; Fung and St John, 1995; Grigson et al., 1998; Sakai and Yamamoto, 1998). The neurons in the pons-midbrain tegmentum that are necessary for wakefulness remain unidentified.

## Human Experiments

In parallel, natural lesion studies have revealed that a similar region of the human brainstem is necessary for wakefulness. Parvizi and Damasio (2003) analyzed patients with brainstem lesions to identify the region necessary for wakefulness, and found that the maximum overlap between coma-causing lesions was in the upper pontine tegmentum (Figure 1G). Fischer et al. (2016) refined this approach by comparing comatose patients to a control group with lesions that did not produce coma and found that comatose patients were significantly more likely to have damage to a focal area of the pontine tegmentum in or near the medial PB (Figure 1H).

## Forebrain Arousal Nodes

**FIGURE 2:**
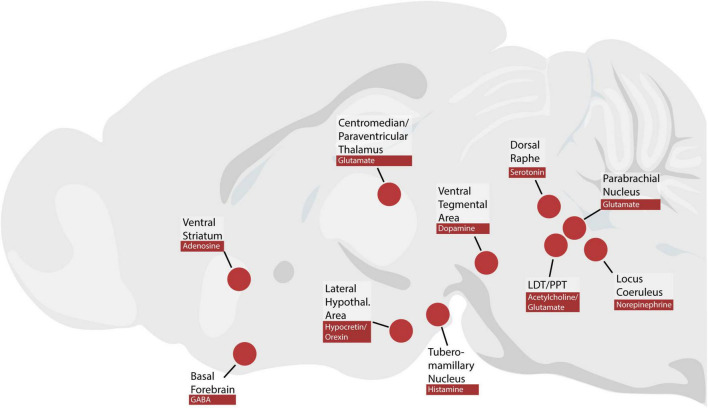
Brain regions and neuromodulators discussed in this review. PPT, pedunculopontine tegmental nucleus; LDT, laterodorsal tegmental nucleus.

The brainstem neurons that maintain wakefulness presumably exert their influence on the forebrain and cause desynchronization of the cortical EEG. The cerebral cortex, when deafferented and left unstimulated, generates slow-wave rhythms reminiscent of sleep (Rinaldi and Himwich, 1955b; Timofeev et al., 2000). Subcortical systems must provide tonic input to keep the cortex in a wake-like rhythm. It remains less clear which subcortical sites relay this input from the brainstem to the cerebral cortex (Figure 2).

### Thalamus

Magoun and colleagues made the influential proposal that the brainstem exerts its awakening influence on the cortex via the thalamus (Moruzzi and Magoun, 1949; Starzl et al., 1951). The thalamus is often viewed as a critical node for arousal circuitry (McCormick and Bal, 1997; Schiff, 2008; Redinbaugh et al., 2020). Electrical stimulation of the thalamus promoted EEG activation (Starzl et al., 1951), and thalamocortical neurons have a role in shaping the cortical EEG signal (Steriade et al., 1993b). However, in a cohort of patients with severe brain injury, arousal level was not correlated with thalamic atrophy or damage (Lutkenhoff et al., 2015). Furthermore, “thalamic” lesions that caused severe loss of consciousness (stupor or coma) extended into the midbrain and pons, and lesions entirely within the thalamus did not eliminate wakefulness (Hindman et al., 2018).

The thalamus is a large structure, and authors have ablated it in experimental animals using a variety of different methods. Extensive electrolytic lesions in cats that destroyed all but the most caudal, medial thalamus caused EEG slowing, but brainstem stimulation still caused EEG activation (Starzl et al., 1951). Villablanca and Salinas-Zeballos (1972) surgically aspirated this region in cats to create the “athalamic” preparation, where the entire thalamus except for the lateral geniculate bodies was destroyed. Although a normal wake-like EEG pattern did not reappear for 10 days, these cats were awake and moving after 1–3 days. Dringenberg and Olmstead (2003) explored whether the brainstem stimulated cortical arousal via the thalamus in rats. Under light urethane anesthesia, brainstem stimulation activated the EEG, and infusing lidocaine to inhibit the basal forebrain caused the cortical EEG to return to sleep-like cortical rhythms. In contrast, infusing lidocaine into the thalamus did not prevent long-lasting cortical activation after brainstem stimulation.

Similarly, unilateral ibotenic acid lesions in the lateral thalamus produced no EEG changes (Buzsaki et al., 1988). To explore whether chronic thalamic lesions increased sleep, Fuller et al. (2011) made extensive ibotenic acid lesions that destroyed all but the most lateral or caudal vestiges of the thalamus. These rats had normal amounts of sleep and wake and largely normal EEG activity, except for a lack of sleep spindles. With the exception of the reticular nucleus, thalamic neurons are glutamatergic (Fremeau et al., 2001; Barroso-Chinea et al., 2007) and using chemogenetics to stimulate glutamatergic thalamic neurons in mice did not increase the time spent awake (Anaclet et al., 2015). However, more recent experiments in mice and monkeys showed that certain midline and intralaminar (paraventricular, centromedian, and centrolateral) thalamic nuclei promote arousal (Gent et al., 2018; Ren et al., 2018; Redinbaugh et al., 2020). These experimental results complement the results of lesion-based symptom mapping, which found an association between injury to the posterior, median region of the human thalamus and mild impairments in arousal (Hindman et al., 2018).

The above lesion experiments challenge the long-held assumption that the thalamus is necessary for arousal. Thalamocortical connectivity is important for the contents of consciousness, and some neurons in the thalamic midline and intralaminar nuclei are capable of enhancing arousal, but the thalamus is not necessary for wakefulness (Hindman et al., 2018). Instead, more recent attention has turned to the basal forebrain and hypothalamus as potential relays required for cortical activation (von Economo, 1930; Nauta, 1946; Fuller et al., 2011).

### Basal Forebrain

To search for extrathalamic pathways that carry ascending activating impulses, investigators stimulated potential subcortical nodes. For example, electrical stimulation of the basal forebrain caused EEG activation in comatose cats with cerveau isolé lesions (1977). Similarly, stimulating the basal forebrain in urethane-anesthetized rats produced arousal (Metherate et al., 1992).

Stimulating the basal forebrain is sufficient to increase arousal, but whether it is necessary depends on how arousal is defined. Extensive kainic acid and electrolytic lesions caused EEG slowing in the ipsilateral cerebral hemisphere, but did not cause coma (Stewart et al., 1984). Similarly, more circumspect ibotenic acid lesions and lidocaine infusions caused EEG slowing (Buzsaki et al., 1988; Dringenberg and Olmstead, 2003) and less rebound sleep after sleep deprivation, but animals remained awake (Kaur et al., 2008). Large lesions generated by injecting orexin-saporin into the basal forebrain caused rats to become comatose after a week (Fuller et al., 2011). To our knowledge, this is the only report of coma after basal forebrain lesions.

Glutamatergic parabrachial neurons that receive input from the sleep-promoting parafacial zone send projections to the basal forebrain, and chemogenetic stimulation of BF-projecting PB neurons increased wakefulness (Anaclet et al., 2014). The basal forebrain contains interspersed cholinergic, glutamatergic, and GABAergic neurons (Gritti et al., 1993; Hassani et al., 2009). Anaclet et al. (2015) used chemogenetic stimulation and inhibition to test the role of each population in wakefulness. Activating the cholinergic population caused a decrease in EEG power below 30 Hz, and activating the glutamatergic population also produced EEG changes, but neither neuronal population altered the time spent awake. Conversely, chemogenetically activating GABAergic neurons produced a large increase in wakefulness and inhibiting them caused a 30% decrease in time spent awake. Photostimulating these parvalbumin-expressing neurons drives 40 Hz gamma band oscillations in the cortex (Kim et al., 2015).

In another study, optogenetic activation of similar groups of neurons produced different results (Xu et al., 2015). They likewise found that activation of parvalbumin-expressing GABAergic neurons produced arousal. Conversely, these authors also found that activating glutamatergic and cholinergic basal forebrain neurons produced arousal. Overall, these results confirm that the basal forebrain modulates EEG rhythm and plays a role in wakefulness.

### Hypothalamus

The hypothalamus was the first brain region implicated in wakefulness. Early evidence came from the outbreak of encephalitis lethargica starting during World War I. After an acute phase of upper respiratory symptoms and fever, patients entered a chronic phase with sleep disturbances. Strikingly, they could fall into “the deepest sopor in which the patients may sleep for weeks and months but from which in the majority of cases, it is possible to rouse them” (von Economo, 1930). Von Economo examined the brains of many of these patients and recognized that patients with lethargy had lesions in the caudal hypothalamus. Conversely, patients with insomnia had damage in the rostral hypothalamus (Berger and Vilensky, 2014). Nauta performed the first experiments testing von Economo’s hypothesis by making knife-cut lesions in the rat hypothalamus and observed similar results (1946). Similarly, large electrolytic lesions in the caudal hypothalamus in monkeys replicated the chronic lethargy and hypersomnolence (Ranson, 1939). However, cell-body specific lesions with ibotenic acid only produced temporary hypersomnolence (Denoyer et al., 1991), suggesting an additional role for axons passing through the posterior hypothalamus (Magoun, 1950).

The lateral hypothalamus is implicated in other functions that may affect wakefulness, such as thermoregulation and feeding (Satinoff and Shan, 1971; Jennings et al., 2013). Therefore, cell-type-specific techniques are necessary to target subpopulations of neurons in this area. Stimulating GABAergic neurons in this area caused wake but inhibiting them did not cause prolonged somnolence (Venner et al., 2016). Two other populations of wake-promoting neurons in the posterior hypothalamus, the hypocretin/orexin neurons and histaminergic neurons of the tuberomammillary nucleus, are discussed below.

## Special Neurotransmitters

Many investigators have postulated that particular neurotransmitters play a special role in arousal (Phillis and Chong, 1965; Jouvet, 1969, 1972; Jones, 2003, 2008; Saper et al., 2010). These neurotransmitters include hypocretin/orexin, histamine, norepinephrine, serotonin, dopamine, adenosine, and acetylcholine, which are produced in separate populations of neurons, some of which are in the brainstem (Figure 2). Much of the evidence for the role of each neurotransmitter consists of pharmacological manipulations, such as infusing exogenous neurotransmitter, receptor agonists, or receptor antagonists. In some cases, subsequent cell-type specific manipulations have challenged these hypotheses.

### Hypocretin/Orexin in the Lateral Hypothalamus

Narcolepsy is a disease characterized by sleep-wake state instability. Although narcoleptics sleep a normal amount, their sleep is fragmented, with intrusion of REM sleep into wakefulness. The classic form, type 1 narcolepsy, is additionally characterized by cataplexy and undetectable levels of orexin in cerebrospinal fluid (Scammell, 2015).

Two complementary studies made it clear that hypocretin/orexin neurons are critical to the pathophysiology of type 1 narcolepsy. One study identified a hypocretin/orexin receptor mutation in narcoleptic dogs, and the other identified cataplexy in hypocretin/orexin knockout mice (Chemelli et al., 1999; Lin et al., 1999). This neuropeptide is produced by neurons in the lateral hypothalamus. Replacing or infusing hypocretin/orexin can ameliorate symptoms in narcoleptic animals (Mieda et al., 2004; Kantor et al., 2013; Kaushik et al., 2018). Infusing it into the brain prolongs wakefulness (Huang et al., 2001). Conversely, hypocretin/orexin receptor antagonists promote sleep and cataplexy in narcoleptic mice (Black et al., 2013) and are used clinically to treat insomnia (Cox et al., 2010; Herring et al., 2012). Hypocretin/orexin-containing axons and receptors concentrate in several arousal-implicated sites, including the tuberomammillary nucleus, dorsal raphe nucleus, and locus coeruleus (Peyron et al., 1998; Marcus et al., 2001), and receptor activation in the posterior hypothalamus may be particularly important for enhancing wakefulness (Mochizuki et al., 2011). However, despite its well-accepted role in promoting and consolidating wakefulness, the largely normal amount of wakefulness in hypocretin/orexin-deficient animals and patients shows that the basic mechanisms of arousal do not require this neuropeptide or the neurons that produce it (Broughton et al., 1988; Mochizuki et al., 2004).

### Histamine in the Posterior Hypothalamus

Drugs that target histamine and its receptors have strong effects on wakefulness. For example, first-generation antihistamine drugs are notable for their sleep-inducing effects, but inhibit both peripheral and central H_1_ receptors and muscarinic acetylcholine receptors (Kubo et al., 1987). They do not reduce wakefulness in H_1_ receptor knockout mice (Wang et al., 2015). Furthermore, the H_3_ antagonist pitolisant is approved for treating excessive daytime sleepiness in narcolepsy (Lin et al., 1990). This drug is thought to boost histamine release by blocking this autoinhibitory receptor on histaminergic neurons (Arrang et al., 1987).

In the brain, histamine is produced in the posterior hypothalamus, primarily in the tuberomammillary nucleus (Watanabe et al., 1984; Valko et al., 2013). Centrally infusing histamine causes EEG activation (Monnier et al., 1970) and behavioral arousal (Kalivas, 1982). Histidine Decarboxylase (HDC) catalyzes the conversion of histidine to histamine and is a specific marker for histaminergic neurons.

Lin et al. (1988) tested the necessity of histamine for arousal with a suicide inhibitor of HDC, α-fluoromethylhistidine (Watanabe et al., 1990). Injecting α-fluoromethylhistidine intraperitoneally and intrahypothalamically caused only a minor decrease in wakefulness (Lin et al., 1988). A similarly small decrease in wakefulness was found in HDC knockout mice (Ohtsu et al., 2001), which cannot produce histamine (Parmentier et al., 2002). These mice also fell asleep sooner after stressful stimuli.

Although histamine itself may not play a necessary role in arousal, the histaminergic neurons of the tuberomammillary nucleus express other neurotransmitters that might. Two groups have used different techniques to explore the roles of these neurons in arousal, with divergent results. Yu et al. (2015) found that HDC-expressing neurons express the GABA synthetic enzyme *Gad1* and reported previous expression of the vesicular GABA transporter *Vgat*. siRNA knockdown of *Vgat* and scrambling of the *Vgat* gene in these neurons markedly increased wakefulness. Conversely, Venner et al. (2019) found that adult *Hdc*-expressing neurons do not express *Vgat* mRNA. Chemogenetic activation prolonged wakefulness after a stressor, but optogenetically silencing these neurons did not alter baseline arousal.

### Norepinephrine in the Locus Coeruleus

There is substantial pharmacological evidence that norepinephrine increases arousal. Peripherally infusing norepinephrine, a hormone that crosses the blood-brain barrier, increased wakefulness in cats (Bonvallet et al., 1954; Rothballer, 1956). Centrally administered norepinephrine likewise activated the EEG in rabbits (Matsuda, 1968, 1969). Infusing norepinephrine, the β-adrenergic agonist isoproterenol, or the α_1_-adrenergic agonist phenylephrine directly into the ventricles or basal forebrain caused arousal in rats (Berridge and O’Neill, 2001).

Attention has therefore focused on the locus coeruleus, which is the major source of forebrain norepinephrine. The axons of the locus coeruleus terminate diffusely through the entire central nervous system, suggesting a role in modulating a global process such as arousal (Jones and Moore, 1977b). The firing rate of putative noradrenergic neurons is highest when animals are awake (Hobson et al., 1975). Infusing the α_2_ adrenoreceptor agonists clonidine or dexmedetomidine into the locus coeruleus inhibited these neurons and caused an anesthetized state (Svensson et al., 1975; Correa-Sales et al., 1992; Berridge et al., 1993). Furthermore, optogenetically stimulating locus coeruleus neurons causes immediate arousal from sleep (Carter et al., 2010).

However, directly inhibiting locus coeruleus and its axons only causes a small and variable reduction in arousal. Injecting the catecholamine neurotoxin 6-OHDA into the central tegmental tract (Lidbrink, 1974) reduced activity but not wakefulness. Dopamine β-hydroxylase (DβH)-knockout mice had a global lack of norepinephrine and epinephrine, and most die *in utero* (Thomas et al., 1995). However, treatment with a norepinephrine precursor that does not require DβH during gestation allowed these mice to survive. One group reported that adult DβH-knockout mice had normal sleep-wake cycles (Hunsley and Palmiter, 2003), while another reported that their waking bouts are shorter, with overall 2 h less wake per day (Ouyang et al., 2004).

Administration of the locus coeruleus toxin DSP4 [N-(2-chloroethyl)-N-ethyl-2-bromobenzylamine], which eliminates most noradrenergic axons from the cerebral cortex (Fritschy and Grzanna, 1991), only caused slight changes to the circadian cycle and did not reduce wakefulness (Gonzalez and Aston-Jones, 2006). Acute, cell-type specific inhibition of locus coeruleus neurons with halorhodopsin only caused a small decrease in wakefulness during the dark period (Carter et al., 2010). Lastly, there is an extensive literature on non-specific locus coeruleus lesions without coma (Bias et al., 1975; Nassif et al., 1983; Lategan et al., 1990; Szot, 2017).

Rather than baseline wakefulness, the locus coeruleus may prolong wakefulness after stimulation. DβH-knockout mice fall asleep sooner after mild stress than control mice (Hunsley and Palmiter, 2003; Ouyang et al., 2004). Gompf et al. (2010) injected anti-DβH-saporin intraventricularly in rats, which caused extensive locus coeruleus cell loss. These rats spent less time awake after novel objects were placed in their cages during the light phase but had normal baseline wakefulness. In conclusion, while norepinephrine administration and locus coeruleus activity can stimulate arousal, they are not necessary for wakefulness.

### Serotonin in the Dorsal Raphe

Initially, serotonin was suspected to inhibit arousal. Much of the literature centered on the use of para-chlorophenylalanine (PCPA), which inhibits serotonin synthesis (Koe and Weissman, 1966) and causes insomnia (Delorme et al., 1966; Weitzman et al., 1968; Borbely et al., 1981). Most forebrain-projecting serotonergic neurons are located in the dorsal raphe nucleus. Electrolytic lesions in this area made cats acutely insomniac (up to 90% wakefulness), and the amount of sleep correlated inversely with the amount of cerebral serotonin (Jouvet, 1969).

Genetically targeted experiments produced different results. Using *Pet1-Cre* to selectively excise floxed alleles of the *Lmx1b* gene (Lmx1b^flox/flox^;ePet-Cre mice), which is required for the differentiation and survival of serotonergic neurons, produced mice with virtually no serotonin in the central nervous system (Zhao et al., 2006). Like cats with dorsal raphe lesions, these mice spent more time awake than controls. However, they were also hypothermic due to impaired thermogenesis (Hodges et al., 2008). When housed at a thermoneutral temperature (33 °C), they had normal sleep-wake cycles (Buchanan and Richerson, 2010). In mice, PCPA likewise caused insomnia at room temperature but not at thermoneutral temperatures (Murray et al., 2015).

Other experiments have led to contradictory conclusions. In support of the possibility that serotonergic neurons promote arousal, hour-long optogenetic stimulation of the dorsal raphe at 20 Hz increased wakefulness (Ito et al., 2013). In contrast, ablation of the serotonergic neurons in the midbrain raphe with a Cre-dependent toxin moderately increased the amount of time spent awake (Oikonomou et al., 2019). Other groups found no change in baseline sleep with a similar ablation strategy (Kaur et al., 2020). Further, chemogenetically activating dorsal raphe serotonergic neurons caused mice to spend slightly more time asleep. Their sleep after i.p. injection of the DREADD ligand clozapine-N-oxide (CNO) was similar to that of uninjected control mice, suggesting that serotonergic neurons in the dorsal raphe nucleus reduce anxiety caused by the injection. Consistent with this, chemogenetic activation also increased the amount of time these mice spent in the middle of an open-field test (Venner et al., 2020).

### Dopamine in the Ventral Tegmental Area and Dorsal Raphe

Dopamine is produced by neurons in a series of catecholaminergic populations in the brainstem. The largest and most well-known of these are the substantia nigra and ventral tegmental area in the ventral midbrain. These dopaminergic neurons are important for movement and motivation, both of which are inextricably linked to arousal, and several lines of evidence point to an important role for dopamine in wakefulness.

Patients with Parkinson’s disease have extensive degeneration of these dopaminergic neurons (Alberico et al., 2015) and often have disorders of sleep and wakefulness (Chahine et al., 2017). Up to 50% of patients suffer from excessive daytime sleepiness (Arnulf, 2005; Videnovic et al., 2014).

There is also substantial pharmacological evidence that dopamine contributes to arousal (Espana and Scammell, 2011). Methylphenidate and other amphetamines, which are widely prescribed, increase extracellular dopamine and promote arousal (Jones et al., 1998; Murnane et al., 2013). Like amphetamines, the wake-promoting drug modafinil blocks the dopamine reuptake transporter (DAT), cannot promote arousal in mice lacking this transporter (Wisor et al., 2001), and requires D1 and D2 receptors to promote arousal (Qu et al., 2008).

Additional animal experiments support an important role for dopamine in arousal. Dopamine-deficient mice, which have no endogenous ability to produce dopamine, are normal at birth, but are grossly hypoactive and soon die without treatment. However, when these mice were injected with levodopa, they synthesized dopamine and survived (Zhou and Palmiter, 1995; Szczypka et al., 1999). Without levodopa, dopamine-deficient mice were still awake, responded to stimuli, and despite low motivation displayed a variety of spontaneous behaviors. However, we lack chronic EEG recordings from these mice, so it is unclear whether they have a normal amount of wakefulness. Dopamine also can be depleted pharmacologically, using α-methyl-p-tyrosine (αMT), a non-specific inhibitor of tyrosine hydroxylase (TH), which eliminates dopamine and a significant amount of norepinephrine (Sotnikova et al., 2005). Mice treated with αMT were behaviorally awake despite hippocampal local field potentials typical of non-REM sleep. Interestingly, these mice never entered REM sleep (Dzirasa et al., 2006).

If dopamine has a role in arousal, which dopaminergic neurons and projections are important? A group of dopaminergic neurons in the periaqueductal gray has historically been considered an extension of the larger A10 dopaminergic population in the ventral tegmental area (VTA) (Lu et al., 2006a). Injecting the catecholamine neurotoxin 6-OHDA into this area caused a loss of ∼60% of these neurons and reduced wakefulness by 20% in rats (Lu et al., 2006a). Chemogenetic inhibition of these neurons also reduced wakefulness by 35% (Cho et al., 2017).

The largest dopaminergic populations in the brain are the substantia nigra and adjacent VTA. Large electrolytic lesions in this area impaired movement without reducing the amount of time awake as measured by EEG (Jones et al., 1973). Destroying dopaminergic and other ventral midbrain neurons by injecting NMDA or orexin-saporin into the substantia nigra and VTA of cats and rats caused a large and lasting increase in wakefulness (insomnia), not coma (Lai et al., 1999; Gerashchenko et al., 2006).

Modern cell-type-specific techniques have allowed researchers to more selectively determine the role of dopaminergic neurons in the VTA. Acutely inhibiting these neurons with chemogenetics caused a large decrease in arousal. Midbrain dopaminergic neurons seem especially important in generating wakefulness after salient stimuli; when these neurons were inhibited, mice spent much less time awake after being presented with palatable food, female mice, or predator odor. Finally, optogenetically activating midbrain dopaminergic neurons induced wakefulness (Eban-Rothschild et al., 2016). Pretreating mice with a D2/D3 receptor antagonist abolished the wake-promoting effect of activating these midbrain dopamine neurons (Oishi et al., 2017).

The dorsal raphe and ventral tegmental area dopaminergic neurons are spatially distinct but seem to have similar activity and functions. Both groups are activated by salient stimuli: predator odors, female mice, and palatable food (Eban-Rothschild et al., 2016; Cho et al., 2017). Optogenetic activation of both groups promotes wakefulness, while chemogenetic inhibition of both groups causes approximately a 30% decrease in baseline arousal and a decrease in arousal when presented with female mice or predator odors. These results may be similar because these groups arise from similar embryologic precursors, and it remains to be seen if inhibiting both groups have an additive effect on baseline arousal.

### Adenosine in the Ventral Striatum

The most widely used wake-promoting drug, caffeine, works by antagonizing adenosine receptors (Snyder et al., 1981). Caffeine specifically boosts arousal by antagonizing A_2A_ receptors (Spealman, 1988; Huang et al., 2005), and deleting these receptors from the nucleus accumbens abolishes the wake-promoting effects of caffeine (Lazarus et al., 2011). Conversely, adenosine receptor agonists increase slow wave EEG activity and sleep when administered at thermoneutral temperatures (Snyder et al., 1981; Dunwiddie and Worth, 1982; Benington et al., 1995; Scammell et al., 2001). Infusion of adenosine itself in animals can cause hypothermia and sleep (Feldberg and Sherwood, 1954; Buday et al., 1961).

A_2*A*_ receptors are co-expressed with D_2_ dopamine receptors by enkephalin-expressing indirect medium spiny neurons in the striatum (Le Moine et al., 1990; Schiffmann et al., 1991). They form a heteromer, where stimulation of the A_2A_ receptors causes reduced effectiveness of the linked D_2_ receptor (Ferre et al., 1991; Canals et al., 2003). It has therefore been postulated that inhibition of adenosine receptors by caffeine causes an increase in dopamine-induced locomotion and arousal (Ferre et al., 1992).

### Acetylcholine in the Brainstem

Acetylcholine is thought to play at important role in arousal (Jones, 2008; Murillo-Rodriguez et al., 2012; Iwanczuk and Guzniczak, 2015; Schwartz and Kilduff, 2015; Saper and Fuller, 2017). Much of the evidence comes from early pharmacological studies.

Placing acetylcholine directly on the cortex (Miller et al., 1940) or in the carotid circulation (Bremer and Chatonnet, 1949) produced arousal. This effect was potentiated by a cholinesterase inhibitor and blocked by atropine, muscarinic receptor antagonist (Miller et al., 1940; Rinaldi and Himwich, 1955a; Stewart et al., 1984). Cholinesterase inhibitors also produced arousal when administered peripherally (Bradley, 1953). Acetylcholine can even produce EEG activation after cerveau isolé (midbrain) transections, but not when placed on the deafferented cortex, suggesting the action of acetylcholine is in a subcortical region of the forebrain (Rinaldi and Himwich, 1955b).

More evidence for acetylcholine in wakefulness comes from the well-studied effects of cholinergic antagonists. Atropine causes EEG slowing in experimental animals when infused peripherally (Schallek and Smith, 1952; Wikler, 1952; Bradley and Elkes, 1957; Domino and Hudson, 1959) or centrally (Rinaldi and Himwich, 1955a; White et al., 1961), but animals remain awake and excitable (Wikler, 1952; Bradley and Elkes, 1957). Similarly, peripherally infusing atropine in humans causes drowsiness and coma at high doses (Longo, 1966). At lower doses, atropine causes a dissociation between EEG and behavioral measures of arousal (Ostfeld et al., 1960). Recipients had depressed mood, slowed EEG activity, and impaired ability to conduct complex mental tasks, but they remained awake and able to converse.

Cholinergic neurons span the brain, and include cranial nerve nuclei, basal forebrain, cortex, and striatum (Armstrong et al., 1983; Mesulam et al., 1983; Zaborszky et al., 2008). They also form two major cholinergic nuclei in the brainstem: the pedunculopontine tegmental nucleus (PPT) and laterodorsal tegmental nucleus (LDT). Because these neurons overlap the region of the brainstem necessary for wakefulness as described above, they are thought to be important for maintaining wakefulness (Steriade et al., 1990; Andrews et al., 2019). Although there is significant evidence for acetylcholine’s role in arousal, the evidence does not support a major role for cholinergic brainstem nuclei.

To examine their role in arousal, a variety of attempts have been made to silence PPT and LDT neurons. Complicating this matter is that cholinergic, glutamatergic, and GABAergic neurons are intermixed in this area of the brain (Ford et al., 1995; Wang and Morales, 2009). Excitotoxic lesions in the mesopontine tegmentum reduced REM sleep without changing the total amount of wakefulness (Webster and Jones, 1988). The number of remaining cholinergic neurons in the PPT correlated with amount of REM sleep but not wakefulness. In another study, small PPT lesions had no effect on overall wakefulness (Petrovic et al., 2013). Larger excitotoxic lesions of the PPT moderately decreased wakefulness, while lesions of the LDT did not (Lu et al., 2006b).

Although high frequency stimulation in this region caused cortical activity reminiscent of arousal (Steriade et al., 1993a), subsequent cell-type specific stimulation of cholinergic neurons did not boost wakefulness. Optogenetic stimulation of these cholinergic neurons induced non-REM to REM transitions, but only had a mild effect on the probability of waking (Van Dort et al., 2015). Furthermore, chemogenetic activation of cholinergic PPT neurons did not change wakefulness but decreased EEG delta power during sleep. Conversely, stimulating glutamatergic neurons in this region increases wakefulness (Kroeger et al., 2017). Together, these results indicate that cholinergic PPT and LDT neurons affect cortical activity but are not necessary for wakefulness.

## Conclusion

There is a striking difference between coma after midbrain transections and the lesser deficits in arousal after cell-type-specific ablation or inhibition (Buchanan and Richerson, 2010; Carter et al., 2010; Kaur et al., 2013, 2020; Eban-Rothschild et al., 2016; Cho et al., 2017; Kroeger et al., 2017; Venner et al., 2019). Bremer, Lindsley, Batini, and others made crude transections that disconnected the brainstem from the forebrain and observed coma and EEG slowing for several days. With the exception of massive brainstem lesions (Lindsley et al., 1950; French and Magoun, 1952; Pais-Roldan et al., 2019), the only report of focal lesion-induced coma in experimental animals we are aware of was that of Fuller et al. (2011). It is unclear why brainstem lesions in this study produced far greater effects than lesions in this area in other studies (Jones et al., 1977; Ohman and Johnson, 1986; Reilly et al., 1993; Sakai and Yamamoto, 1998). It will be important to replicate and extend this work with cell-type specific methods.

What can explain the difference between coma caused by brainstem transection and the more modest reduction in wakefulness caused by cell-type specific inhibition? One hypothesis is that many cell groups work in concert to produce wakefulness (Saper et al., 2005; Franks, 2008; Saper and Fuller, 2017; Scammell et al., 2017). In this view, wakefulness is an emergent property of a synergistic, interconnected network of brainstem neurons. Damage to any part of this system causes at most a small or transient decrease in wakefulness, but damage to the entire system is required to produce coma.

Another possible explanation is that some undefined set of upper-brainstem neurons sustains wakefulness. These neurons may or may not have been already described, but their role in arousal has not yet been tested. Under this hypothesis, when this group of cells is completely disconnected from the forebrain, coma results. Other neurons still may promote arousal in certain circumstances and eliminating them would impair a specific mode of arousability. For example, dopamine promotes arousal, and inhibiting dopaminergic neurons in the VTA reduces arousal to salient stimuli, which may account for a small portion of total wakefulness (Eban-Rothschild et al., 2016).

Two crucial results support this undiscovered-neuron hypothesis. First, transection studies reported a sharp loss of wakefulness as lesions advanced rostrally past the pons-midbrain junction, rather than a gradual reduction of wakefulness. Placing transections within a few millimeters in the cat brainstem is the difference between a normal EEG and a comatose one (Rossi and Zirondoli, 1955; Batini et al., 1959). Second, human coma-causing lesions likewise localize to a small area of the upper brainstem tegmentum. These findings support the idea that a focal group of neurons in this area maintains wakefulness (Parvizi and Damasio, 2003; Fischer et al., 2016). Presumably, neurons here exert their influence via output connectivity to arousal centers in the forebrain, including the basal forebrain and cerebral cortex.

As noted above, silencing specific neuronal populations once thought to sustain wakefulness have produced results well short of coma. Inhibiting the serotonergic dorsal raphe, cholinergic pedunculopontine tegmental nucleus or histaminergic tuberomammillary nucleus produced little or no change in sleep/wake amounts (Kroeger et al., 2017; Venner et al., 2019, 2020). Inhibiting the noradrenergic locus coeruleus, dopaminergic periaqueductal gray, or the dopaminergic ventral tegmental area produced mild decreases in wakefulness (Ouyang et al., 2004; Eban-Rothschild et al., 2016; Cho et al., 2017). Among the nuclei hypothesized to have a role in arousal, the necessity of parabrachial neurons has not been exhaustively tested using cell-type specific techniques.

There are two less likely explanations for the contrast between the results of brainstem transection and focal inhibition. First, species differences in wakefulness mechanisms could help explain the smaller effects of cell-type-specific manipulations in mice. After midbrain transection, rats (Hanada and Kawamura, 1981) appear to recover EEG activity faster than cats (Slosarska and Zernicki, 1973). It is possible that mice recover from coma even more quickly. Most reports of coma come from immediate lesions, rather than slower genetic or toxin-based approaches. It is even possible that wakefulness in mice does not depend on the brainstem; so far, no reports of a focal, coma-causing lesion have appeared in mice.

Another possible explanation is that the brain structures that maintain wakefulness in this region may not be neuronal somata. The trigeminal nerve enters the brainstem at this level, and transections rostral to it deprive the brain of all somatosensory input, which may be the cause of coma (Bremer, 1935; Roger et al., 1956). It is unlikely that transections cause coma by interrupting non-trigeminal somatosensory input, as coma has not been reported with lesions located further caudally (Rossi and Zirondoli, 1955; Posner et al., 2007).

In the century since Bremer began his experiments, vastly improved neuroscience techniques offer opportunities to further develop his discovery. With cell-type-specific techniques, we now have the power to selectively activate, inhibit, lesion, and record genetically defined populations of neurons. Despite the power of these techniques, we still do not have an answer to the fundamental question raised by Bremer’s research: why do cerveau isolé lesions cause coma?

## Author Contributions

FG and JG wrote the first draft of the manuscript. All authors contributed to the study conception and design, commented on following versions of the manuscript, and read and approved the final manuscript.

## Conflict of Interest

The authors declare that the research was conducted in the absence of any commercial or financial relationships that could be construed as a potential conflict of interest.

## Publisher’s Note

All claims expressed in this article are solely those of the authors and do not necessarily represent those of their affiliated organizations, or those of the publisher, the editors and the reviewers. Any product that may be evaluated in this article, or claim that may be made by its manufacturer, is not guaranteed or endorsed by the publisher.
